# An integrative approach combining ion mobility mass spectrometry, X-ray crystallography, and nuclear magnetic resonance spectroscopy to study the conformational dynamics of α_1_-antitrypsin upon ligand binding

**DOI:** 10.1002/pro.2706

**Published:** 2015-07-14

**Authors:** Mun Peak Nyon, Tanya Prentice, Jemma Day, John Kirkpatrick, Ganesh N Sivalingam, Geraldine Levy, Imran Haq, James A Irving, David A Lomas, John Christodoulou, Bibek Gooptu, Konstantinos Thalassinos

**Affiliations:** 1 Institute of Structural and Molecular Biology, Division of Biosciences, Division of Biosciences, University College LondonLondon, WC1E 6BT, United Kingdom; 2 Wolfson Institute for Biomedical Research, Division of Medicine, University College LondonLondon, WC1E 6BT, United Kingdom; 3 Institute of Structural and Molecular Biology, Department of Biological Sciences, Birkbeck College, University of LondonLondon, WC1E 7HX, United Kingdom; 4 Division of Asthma, Allergy and Lung Biology, King's College London, Guy's HospitalLondon, SE1 9RT, United Kingdom

**Keywords:** ion mobility mass spectrometry, protein dynamics, drug discovery, α1-antitrypsin, protein unfolding, mass spectrometry/methods, nuclear magnetic resonance, biomolecular, X-ray crystallography

## Abstract

Native mass spectrometry (MS) methods permit the study of multiple protein species within solution equilibria, whereas ion mobility (IM)-MS can report on conformational behavior of specific states. We used IM-MS to study a conformationally labile protein (α_1_-antitrypsin) that undergoes pathological polymerization in the context of point mutations. The folded, native state of the *Z*-variant remains highly polymerogenic in physiological conditions despite only minor thermodynamic destabilization relative to the wild-type variant. Various data implicate kinetic instability (conformational lability within a native state ensemble) as the basis of *Z* α_1_-antitrypsin polymerogenicity. We show the ability of IM-MS to track such disease-relevant conformational behavior in detail by studying the effects of peptide binding on α_1_-antitrypsin conformation and dynamics. IM-MS is, therefore, an ideal platform for the screening of compounds that result in therapeutically beneficial kinetic stabilization of native α_1_-antitrypsin. Our findings are confirmed with high-resolution X-ray crystallographic and nuclear magnetic resonance spectroscopic studies of the same event, which together dissect structural changes from dynamic effects caused by peptide binding at a residue-specific level. IM-MS methods, therefore, have great potential for further study of biologically relevant thermodynamic and kinetic instability of proteins and provide rapid and multidimensional characterization of ligand interactions of therapeutic interest.

PDB Code(s): 4PYW

## Introduction

Aberrant conformational behavior of proteins during and after folding is recognized as the basis for an increasing number of chronic diseases, including Alzheimer's, Parkinson's, and Huntington's disease.^1^ α_1_-Antitrypsin deficiency is a conformational disease associated with severe lung (early-onset, panacinar emphysema) and liver (hepatic cirrhosis, hepatocellular carcinoma) disease.^2^,^3^ The *Z* (Glu342Lys) mutation causes α_1_-antitrypsin, the major circulating human antiprotease, to misfold and self-associate into polymers within the endoplasmic reticulum of hepatocytes, with toxic gain-of-function effects.^4^–^6^ The concomitant deficiency of circulating protein renders lung tissue vulnerable to destructive and proinflammatory effects of neutrophil elastase, that is the physiological target of α_1_-antitrypsin, and hence a clinical association with early-onset emphysema.^7^–^9^ In populations of North European descent, the heterozygote frequency for the *Z*-variant is as high as 1 in 27.^10^ Although the risk of severe disease is strongly associated with the homozygous state, α_1_-antitrypsin deficiency is one of the most common monogenic disorders. It remains the only genetic cause of chronic obstructive pulmonary disease identified to date. The clinical need remains largely unmet: no specific treatment other than organ transplantation has proven robustly effective in stabilizing the associated liver or lung disease.^11^ Its disease mechanisms, however, are among the best characterized of any human disease, and hence extensive efforts are underway to translate these scientific insights into novel therapeutic strategies.^11^,^12^

The general process of α_1_-antitrypsin polymerization is driven by thermodynamic considerations. It proceeds from a metastable native state, via an unstable monomeric intermediate state that is polymerogenic (M*), to a hyperstable polymer assembly.^13^,^14^ The potential for this is inherent in α_1_-antitrypsin and other members of the serpin (serine protease inhibitor) protein superfamily as transition from a metastable to a hyperstabilized (enzyme complexed) state underlies the functional mechanism.^15^ It was therefore hypothesized that disease mutations were polymerogenic primarily as a consequence of thermodynamic destabilization of the native state.^14^ This would lower the activation energy of M* formation, favoring its population and polymerization. Indeed, polymerogenic mutations do tend to reduce the thermodynamic stability of the native state, and therefore this is likely to underlie some of the polymerization tendency of deficiency variants. However, the relevance of this to *Z* α_1_-antitrypsin deficiency may have been overstated previously as readouts from assays that were used to report the destabilization of the native fold also reported on polymerization.^16^

An alternative mechanism that has been proposed to underlie the polymerogenic behavior of *Z* α_1_-antitrypsin is that of increased conformational lability (kinetic instability) induced by the mutation.^17^ This is best considered in the context of the native ensemble in solution in which some of the conformers can induce and participate in polymerization. Disease mutations may therefore alter the kinetics of interchange within the ensemble, favoring population of the polymerogenic conformers relative to the wild-type protein. As polymerization is an essentially irreversible process, and therefore under kinetic control,^16^ the propensity to polymerize is partially uncoupled from the thermodynamic stability of the overall fold, as supported by experimental data for a nonglycosylated form of *Z* α_1_-antitrypsin.^17^ This phenomenon is also seen in other polymerizing systems.^18^,^19^

Induced has implications for strategies to identify new therapies to treat *Z* α_1_-antitrypsin deficiency. Most scalable methods for the screening of potential small-molecule ligands against a protein target are geared toward readouts reporting changes in thermodynamic stability. Ion mobility (IM)-mass spectrometry (MS), however, offers the possibility of quantifying the conformational lability of proteins in their native state. IM-MS can separate coexisting forms of the same protein that would otherwise be indistinguishable using MS alone.^20^ The time it takes an ion to traverse the IM cell is related to its mass, charge, and rotationally averaged collision cross-section (CCS), the latter being a measure of the overall shape of the ion.^21^,^22^ By calculating the spread of the CCSs for species generated by soft-ionization techniques (predominantly nanoelectrospray) that preserve the native state of a protein, different conformers can be separated and relatively quantified.^23^ In addition, subjecting ions to increasingly higher collision energies and monitoring the resulting conformations using IM-MS can be used to monitor unfolding pathways of these ions and how such pathways are affected by ligand binding.^24^

We therefore chose to study whether IM-MS could be applied to detect ligand-induced changes in the kinetic stability of α_1_-antitrypsin. To provide complementary information we undertook, in parallel, structural studies using X-ray crystallography and nuclear magnetic resonance (NMR) spectroscopy. All three methods were used for the detailed characterization of the interaction of α_1_-antitrypsin with the reactive loop analogue tetrapeptide Thr-Thr-Ala-Ile (TTAI), the most promising polymerization-blocking peptide developed to date.^25^ This is believed—but has not been definitively shown—to target a critical site for polymer formation (strand 4, β-sheet A: s4A). This site can accommodate a β-strand of approximately 12 residues in length. To date, the interaction has been modeled with the part of the site (upper s4A) occupied by partial intramolecular insertion of the reactive site loop, with a TTAI peptide filling the remainder (lower s4A).^25^

## Results

### Biochemical confirmation that kinetic destabilizing effects of *Z* mutation on α_1_-antitrypsin likely drive polymerization *in vivo*

**Figure 1:**
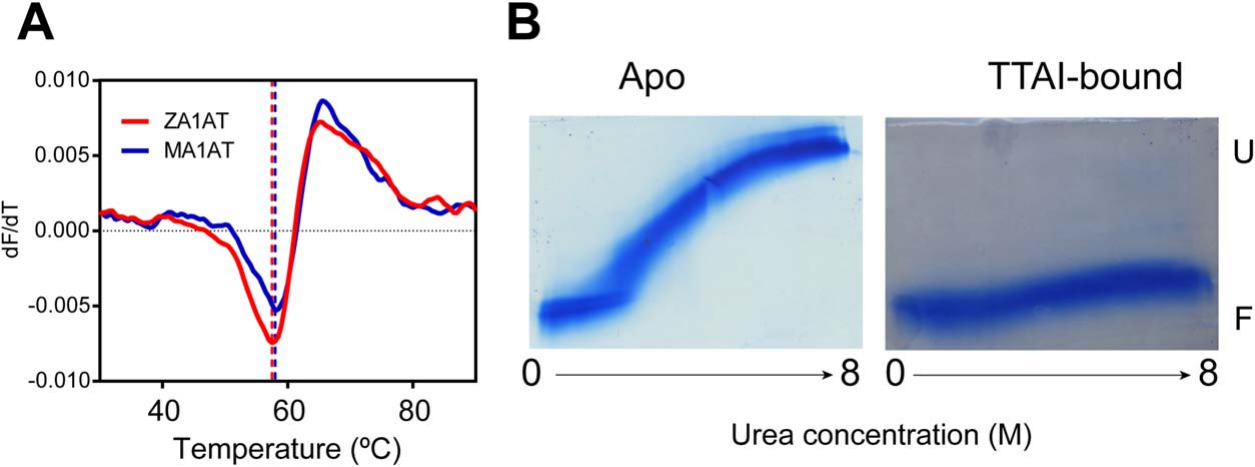
Binding of TTAI to α_1_-antitrypsin. A: Minor thermal destabilization of *Z* relative to *M* α_1_-antitrypsin. Representative traces of observed change in Sypro Orange fluorescence (d*F*/d*T*) during thermal denaturation of *M* and *Z* α_1_-antitrypsin. *T*_m_ values calculated based on the mean of three repeats for *M* and *Z* α_1_-antitrypsin are 59.5 ± 0.3 and 58.5 ± 0.3, respectively. B: Inn brief, 0–8*M* TUG-PAGE of wild-type α_1_-antitrypsin in the apo-state (native conformation, left panel) and after incubation with the TTAI peptide (peptide-complexed, right panel). The native conformer demonstrates characteristic metastability, unfolding from the compact native (folded denoted as F in the figure) state to an extended denatured (unfolded denoted as U in the figure) state across the gradient. Conversely, the TTAI-complexed state demonstrates hyperstability to chemical denaturation that is typical of a reactive loop or an analogous peptide filling the s4A site in serpins.

As a preliminary study, we assessed the thermal stabilities of *ex vivo*, glycosylated wild-type (M) α_1_-antitrypsin and of the *Z* (Glu342Lys) variant that is responsible for the major burden of clinical disease. This was done to address the apparent discrepancy between the reported findings for nonglycosylated recombinant and *ex vivo* α_1_-antitrypsin described above, particularly as its glycosylation occurs cotranslationally.^26^ To avoid the confounding effect of conformational heterogeneity on the methods used previously, we minimized coincident polymerization and aggregation by performing an assay whose readout directly reports transition from the native conformation alone^16^ using low concentrations of protein. In these conditions, the observed *T*_m_ for plasma-derived glycosylated α_1_-antitrypsin was reduced by 1°C in the *Z*-variant relative to the wild-type *M* protein [Fig. 1(A)]. These findings reconcile the previous results in recombinant and *ex vivo* α_1_-antitrypsin. They indicate that, as in the recombinant protein, the disease-relevant behavior of the glycosylated *Z*-variant is predominantly due to the mutation's kinetic rather than thermodynamic effects. Therefore, to properly assess the perturbations to the polymerization mechanism owing to ligand binding, a technique that reports both kinetic and thermodynamic properties is required. This encouraged us to evaluate the potential for native IM-MS methods as a system for the characterization of ligand binding on the stability of α_1_-antitrypsin.

### Stoichiometry of binding probed by native MS

A potent inhibitor of polymerization, TTAI, was originally identified using a semi-rational screening approach^25^; however, its binding mode has not yet been fully characterized. It was thus selected as a suitable molecular tool for the evaluation of native IM-MS techniques in identifying the modulators of conformational stability.

In an initial experiment, wild-type α_1_-antitrypsin in the presence and absence of peptide was probed by 0–8*M* of transverse urea gradient (TUG)-PAGE [Fig. 1(B)]. The apoprotein unfolded at high urea concentrations to give a slow-migrating extended species as reported previously.^27^ The formation of the α_1_-antitrypsin:peptide complex conferred resistance to unfolding in conditions up to 8*M* of urea and therefore ran as a compact species that migrated similarly across the entire urea gradient. By analogy with reactive loop-inserted α_1_-antitrypsin after the interaction with a serine protease,^27^ this stabilized conformation is highly consistent with filling of the cryptic s4A.

**Figure 2:**
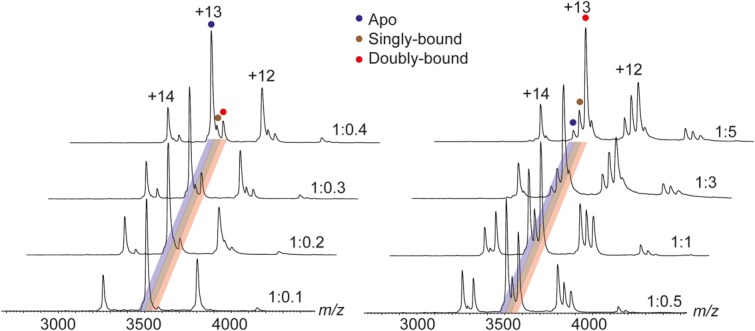
Native mass spectra of increasing TTAI:α_1_-antitrypsin molar ratios. The predominant charge states observed are +12 to +14. Peaks corresponding to the apo-, singly, and doubly bound forms of the protein are highlighted in blue, brown, and red, respectively.

**Figure 3:**
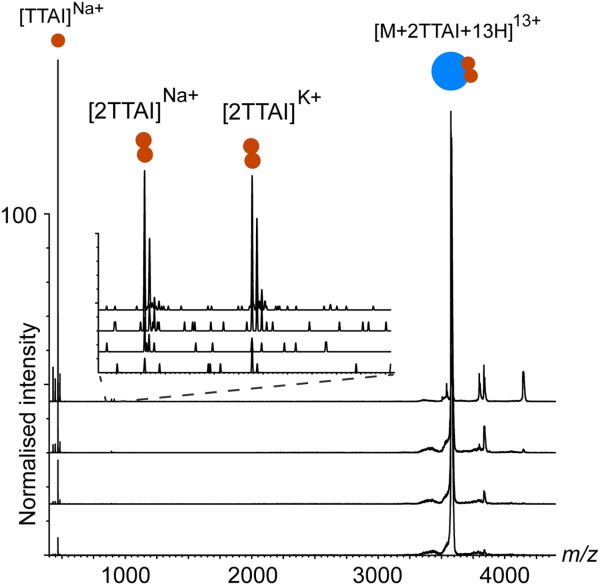
Tandem mass spectra of the +13 charge state corresponding to α_1_-antitrypsin bound to two TTAI molecules. At increasing collision energies (bottom to top), the intensity of the peak corresponding to TTAI increases. Inset shows that at high-collision energies, peaks corresponding to two molecules of TTAI are observed to dissociate from the complex as a pair of directly interacting peptides. This indicates that the two TTAI copies bind to adjacent regions in α_1_-antitrypsin.

To probe the interaction of TTAI with α_1_-antitrypsin further, the protein was incubated with increasing molar amounts of TTAI and analyzed by native MS (Fig. 2). In the absence of TTAI, a narrow charge state distribution from +12 to +14 charge states was observed, indicative of a well-folded protein.^28^ As the amount of the peptide was increased, peaks corresponding to one and two copies of bound TTAI were observed. Notably, even at low peptide concentrations, the intensity of the doubly bound species was higher than that corresponding to the singly bound species. This suggests that binding of TTAI to α_1_-antitrypsin proceeds in a co-operative manner. To further confirm that TTAI was bound to α_1_-antitrypsin, the quadrupole was used to isolate the +13 charge state corresponding to the ternary complex (two copies of TTAI bound), and subjected to collision-induced dissociation (Fig. 3). As the collision voltage was increased, increasing signal corresponding to TTAI, in the form of a sodiated adduct, was observed. Interestingly, peaks corresponding to two copies of TTAI dissociating from the protein in a complex as both sodium and potassium adducts were also observed. This is consistent with the two copies of TTAI binding adjacently within an extended interaction site. Taken together, these data strongly support the binding of two TTAI peptides in the upper and lower parts of the s4A site in α_1_-antitrypsin, with binding of the first peptide facilitating patency of the rest of the site for the second.

### X-ray structure of α_1_-antitrypsin bound to TTAI

**Figure 4:**
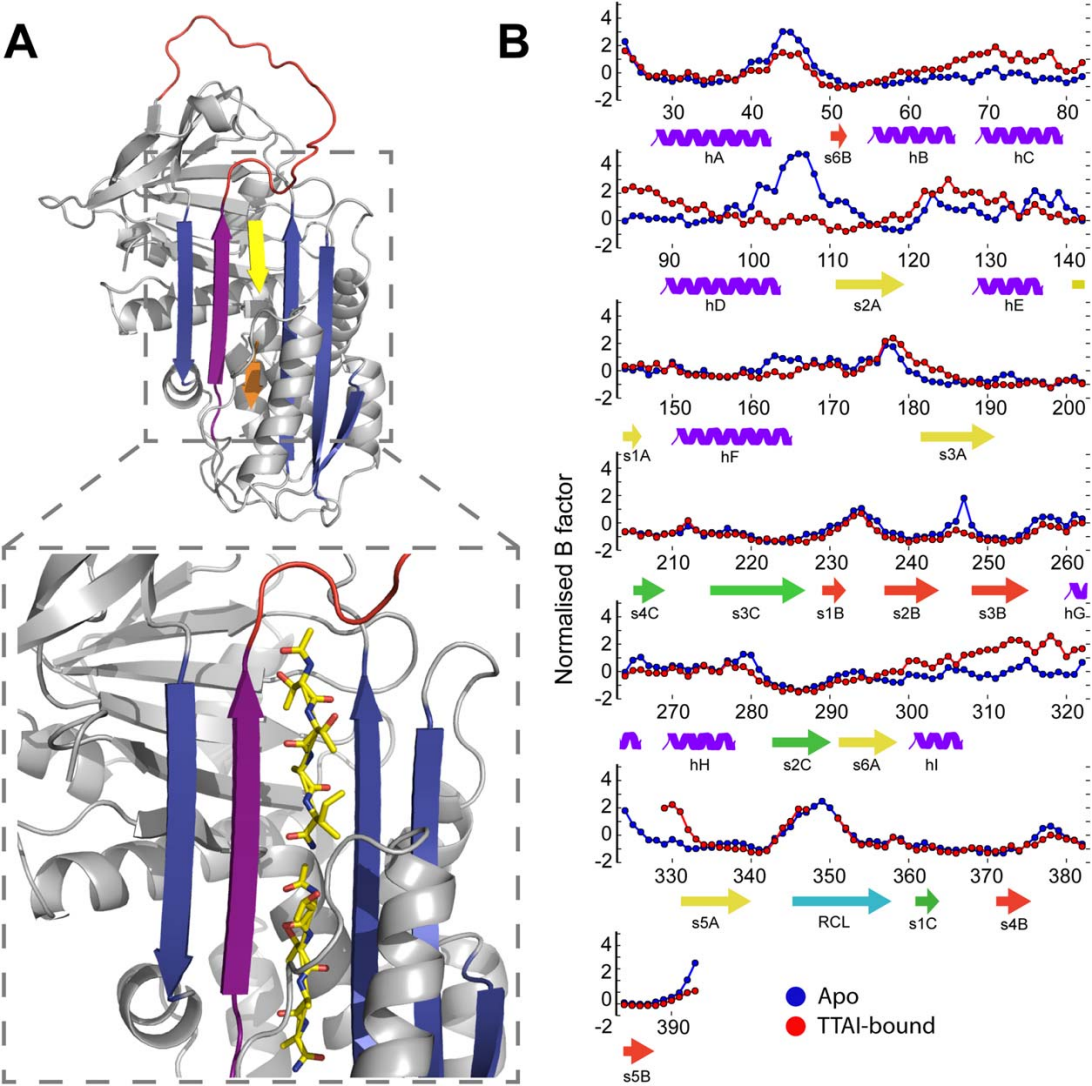
X-ray crystal structure of α_1_-antitrypsin in ternary complex with two copies of the TTAI peptide. A: 1.9 Å X-ray crystal structure of wild-type α_1_-antitrypsin in ternary complex with two copies of the TTAI peptide. PDB ID: 4PYW, with β-sheet A shown in blue, reactive site loop in red, s5A strand in purple, and TTAI peptides in yellow (stick representation). Zoom highlights binding site. B: *B*-factor analysis of high-resolution crystal structures. *B*-factors for each protein were normalized as described previously.^29^ Briefly, the normalized *B*-factor for each residue was calculated by subtracting the average *B*-factor value and dividing by the standard deviation of the *B*-factors for each protein. Only C-alpha atoms were considered for each residue.

Next, we characterized the nature of the TTAI peptide:α_1_-antitrypsin interaction by determining a crystal structure of the ternary complex at a resolution of 1.9 Å (Fig. 4(A), Protein Data Bank [PDB] ID: 4PYW). This confirmed the structural interpretation of the data from TUG-PAGE and initial native MS studies, with two copies of the peptide bound in the s4A site at the center of β-sheet A. The two copies together form an antiparallel β-strand between strands s3A and s5A. This contrasts with the proposed TTAI mode of interaction in which the sheet incorporates a single peptide and a partially inserted reactive center loop.^25^ To gain some insight into the conformational dynamic implications for peptide binding, we carried out *B*-factor analysis for the wild-type and peptide-bound forms of the protein [Fig. 4(B)]. *B*-factors between the two forms of the protein were normalized as previously described by Yuan *et al*.,^29^ so that the distribution of *B*-Factors for each protein had a distribution of zero mean and unit variance. The binding of two copies of TTAI was associated with a decrease in the normalized value for residues between helix D and s2A and an increase for residues between helix I and s5A around the lower peptide insertion site. Away from β-sheet A, RMSD comparisons of the crystallographic positions of backbone and side-chain atoms indicated minimal differences (Supporting Information Figs. 1 and 2).

### NMR analysis of peptide binding

**Figure 5:**
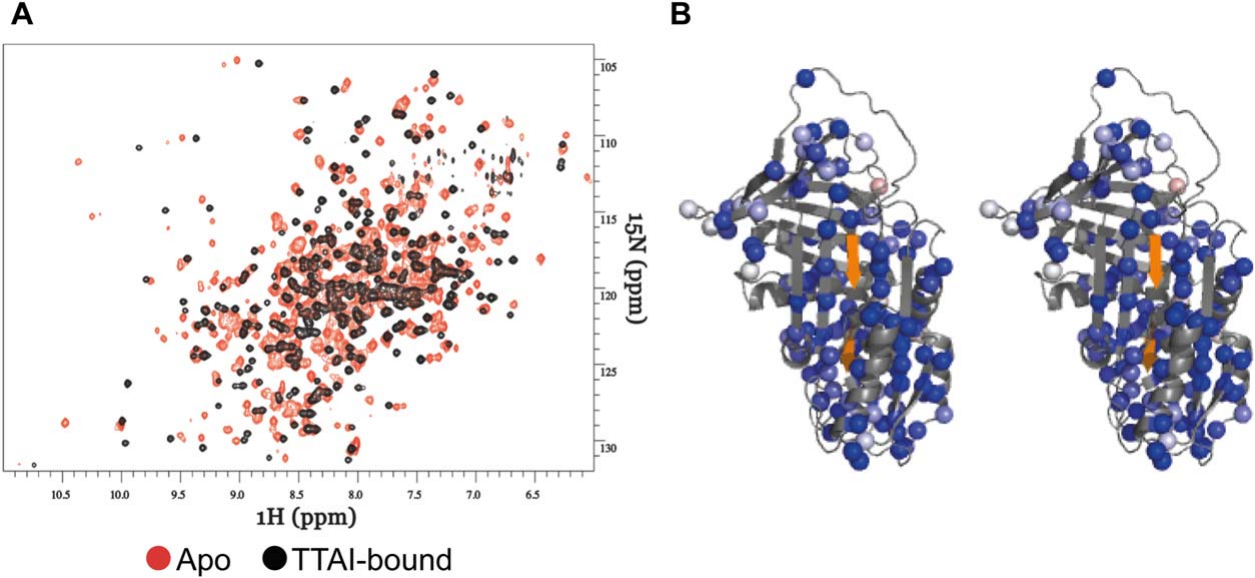
NMR characterization of the structural changes in α_1_-antitrypsin caused by opening of β-sheet A and binding of TTAI peptide. A: ^1^H-^15^N TROSY-HSQC spectra for 5:1 (black) and 0:1 (control, orange) molar ratio TTAI:α_1_-antitrypsin incubations. B: Structural distribution and magnitude of changes reported by β-sheet A opening and TTAI peptide binding in terms of intensity change for the subset of residues that can be unambiguously identified by TROSY-HSQC at 37°C. These residues are shown as colored spheres; all other residues are colored in gray. Heatmap coloring of the crosspeak intensity changes (*I*_5:1_/*I*_0:1_) is shown such that maximal change (complete loss of intensity of initial signal) is shown maximally blue (as it is the case for almost all observable residues [spheres]) and no change *I*_5:1_/*I*_0:1_ = 1.0 in white.

Although *B*-factor analysis can report on the local mobility of the protein within a crystal lattice, the dynamic and structural changes occurring upon peptide binding in solution can be monitored by NMR spectroscopy (Fig. 5). We have previously assigned the entire backbone of α_1_-antitrypsin using 3D NMR spectroscopy.^30^ Alterations in the intensity of crosspeaks in 2D, ^1^H-^15^N TROSY-HSQC spectra, therefore, allowed quantification of the interaction at the residue-specific level. A subset of 102 residues could be unambiguously assigned to crosspeaks in the well-dispersed region of such spectra for the native, apo-form of α_1_-antitrypsin and hence followed across a titration with the TTAI peptide (Supporting Information Fig. 3). As the stoichiometric ratio of TTAI was increased, there was a reduction in the intensities of the native crosspeaks and a concomitant appearance and growth of new crosspeaks (corresponding to the TTAI-bound form), indicating that the binding kinetics are in a slow-exchange regimen on the NMR timescale. Very widespread changes were observed in the spectrum. When mapped to the 3D structures of the apo- or ternary complexed state, these indicated that binding of TTAI affected all structural motifs other than β-sheet C (Fig. 4(B) and Supporting Information Fig. 4). In the saturated ternary complex, the majority of resonances reporting residues in the apo-form of α_1_-antitrypsin were completely lost (i.e., total loss of intensity). In the structural representations, this is indicated by maximal blueness in the red–white–blue heatmap scale, where white corresponds to no change.

### IM-MS collision-induced unfolding analysis of binding

**Figure 6:**
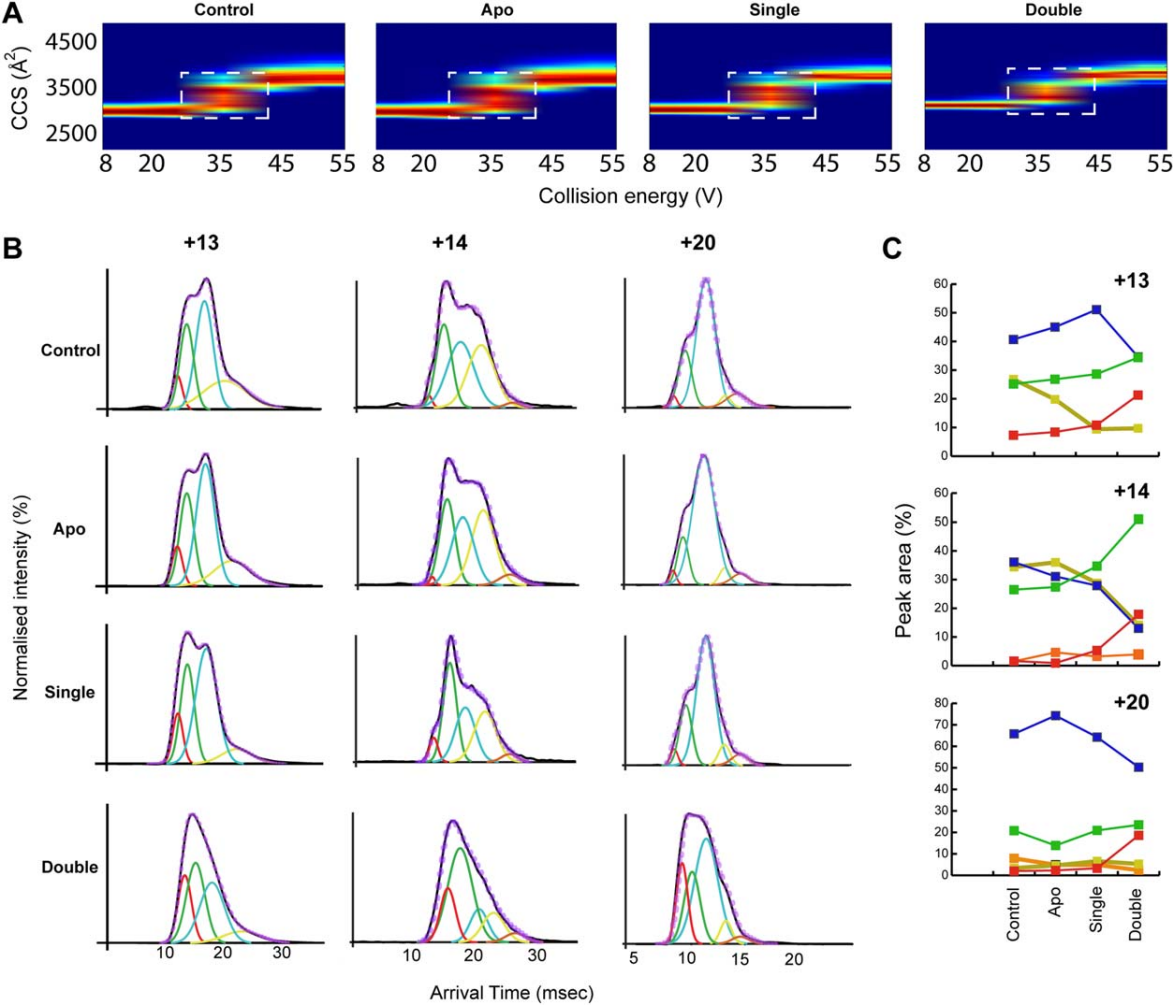
Probing the stability of different peptide-bound states of α_1_-antitrypsin using IM-MS. A: CIU fingerprints generated using Amphitrite for the +13 charge state of α_1_-antitrypsin. The change in CCS (*y*-axis) is plotted against increasing collision energies (*x*-axis) for apo-, singly bound (binary complex with TTAI), and doubly bound (ternary complex) α_1_-antitrypsin to TTAI. A control sample of α_1_-antitrypsin that was not incubated with TTAI peptide was also included (Control). The fingerprint region with the greatest conformational flexibility is highlighted by a white box centring at 35 V. B: Arrival time distributions of the +13, +14, and +20 charge states for the different peptide-bound states of α_1_-antitrypsin. Gaussian peaks representing the conformational species present are fitted to the experimental data, colored in black, with the sum of fitted peaks colored in dashed purple. C: Plot of the peak areas for the fitted Gaussians is shown (B). As the number of bound TTAI molecules increases, so do the areas corresponding to the more compact conformers (red- and green-colored peaks).

Ion mobility coupled to mass spectrometry (IM-MS) has recently emerged as a powerful method for the study of proteins and protein complexes.^31^,^32^ In addition, this method can be extended to probe the structural stability of proteins and their complexes upon small-molecule binding using an approach termed collision-induced unfolding (CIU).^24^ This entails subjecting specific ions to increasing collision energies and characterizing the resulting conformational ensembles using IM-MS. We used CIU to dissect the effects of the distinct binding events to α_1_-antitrypsin. Rather than focusing solely upon extensive unfolding of the polypeptide chain, we assessed whether this approach would exaggerate the differences in kinetic stability between differently bound states. This can be viewed as a “stress test” of conformational behavior of uncomplexed and differentially TTAI complexed α_1_-antitrypsin induced by higher collision energies. Figure 6 shows the results of a CIU experiment performed on the +13 charge state of apo-, single-, and double-bound forms of the protein from a sample of 1:2 protein:peptide mixture. We also performed the CIU analysis on a control sample containing only α_1_-antitrypsin. Figure 6(A) shows the change in CCS as the collision energy increases. At low collision energies, most reflective of nonperturbed conformations, all forms of the protein exist in a single conformational family. The differences between the widths of the CCS peaks are, however, evident. At low collision energies, as the number of bound TTAI molecules increased, the width of the CCS peak decreased, indicating that TTAI binding increases kinetic as well as thermodynamic stability. As a narrow peak width reflects a stabilization of the protein and a reduction of the conformational ensemble present,^33^,^34^ TTAI binding, therefore, increased both kinetic and thermodynamic stability. At increased collision energies, ion collisions with gas within the collision cell cause the protein to unfold. Between 25 and 40 V, a number of coexisting conformers appear for the apo-state, whereas at higher energies the number of these conformers decreases. To see how peptide binding affects these intermediate conformational families, we extracted the arrival time distribution at 35 V collision voltage for charge states +13 and +14 and at 20 V for charge state of +20 [Fig. 6(B)]. Each arrival time distribution was deconvoluted by Gaussian peak fitting and the area under the curve for each of these Gaussians was plotted [Fig. 6(C)]. We examined low charge states (+13 and +14) as previous studies have shown that such charge states are more reflective of structures found in solution.^28^ We also examined a high charge state (+20) as an increase in charge predictably corresponds to a more Coulombically stressed conformational ensemble. Reassuringly, no major differences were observed between control α_1_-antitrypsin that had not been incubated with TTAI, and apo-states of the protein within the mixture of different binding states in the presence of TTAI. This was true for all charge states. Binding of a single TTAI molecule resulted in a small increase in the population of more compact conformers within the ensemble, with a concomitant reduction of the more extended ones. The binding of the second TTAI peptide resulted in a greater increase in the population of more compact conformers relative to the more extended states. At higher collision energies, the differences between apo-, singly bound (binary complex with TTAI), and doubly bound (ternary complex) became more pronounced. A similar trend was observed for the high charge state although the effect of binding a single TTAI peptide is not as noticeable as for the low charge states. This suggests that in higher charge states the destabilizing effects of Coulombic repulsion outweigh the kinetic stabilization caused by binding of one but not two copies of the TTAI peptide.

## Discussion

The structure and dynamics of proteins in solution mediate their behavior in health and disease. These can be related to energy landscapes of the available conformational space determined by sequence and environment^35^ and are well suited to characterization by native IM-MS methods. Local and global energy minima define thermodynamically metastable and stable states, respectively. Slope gradients relate to the kinetics of transitions into these states (folding) and between them (conformational exchange). Pathogenic point mutations in translated polypeptides, or pathological alterations in the environment, can therefore affect either thermodynamic or kinetic determinants of solution behavior or both. Although thermodynamic effects may be more readily quantified, kinetic destabilization has been shown to be important for conformational disease process.^36^

The previous studies of glycosylated α_1_-antitrypsin purified *ex vivo* from human plasma indicated that the *Z* mutation caused a 9°C reduction in *T*_m_. This change, together with the data from other mutants assessed in the same way, suggested that α_1_-antitrypsin polymerization tendency showed a close inverse correlation with thermodynamic stability.^14^ However, in native nonglycosylated α_1_-antitrypsin expressed in *Pichia pastoris*, the observed thermodynamic stability was only mildly reduced by the mutation (1°C reduction in *T*_m_) with kinetic destabilization effects correlating better with polymerogenicity.^17^ This is consistent with the data from the studies in cell models of α_1_-antitrypsin expression in health and disease. Mutations that thermodynamically stabilize α1-antitrypsin by up to 10°C (e.g., Thr114Phe by +5°C, Gly117Phe by +10°C)^37^,^38^ reduce α_1_-antitrypsin polymerization rates at physiological temperatures when introduced on a wild-type background. However, they have no or minimal effect on the polymerization/deficient secretion phenotypes when introduced on the background of the *Z* mutation in cells.

Such data now indicate that the effects of the common, severely pathogenic *Z* allele are mediated more by kinetic rather than thermodynamic destabilization.^17^,^39^ It follows that therapeutic strategies targeted specifically to modulate conformational sampling within the native state ensemble may be beneficial in *Z* α_1_-antitrypsin deficiency. Such an approach has more general potential.^40^ However, at present, compound screens aimed at addressing disease-relevant solution behavior are overwhelmingly assessed by thermodynamic readouts, such as change in thermal or chemical denaturation sensitivity.^12^ It is therefore important to develop alternative methods that report upon the conformational sampling behavior of proteins in solution. In theory, a number of techniques can inform upon protein structure and dynamics. However, if they are to be used in a drug-screening framework, such methods should ideally be highly scalable.

We have compared in parallel NMR spectroscopy, high-resolution X-ray crystallography and native IM-MS approaches to study the structural and dynamic effects of a model peptide representative of a lead compound: α_1_-antitrypsin interaction in solution. TTAI represents the most refined agent developed to date capable of a direct polymerization blockade with impressive effects reported in cell models of disease.^12^,^25^,^41^ In addition to the dramatic thermodynamic stabilization that characterizes the insertion of peptide analogues of the reactive loop in the center of β-sheet A,^42^,^43^ TTAI binding has major effects on the kinetic stability of α_1_-antitrypsin.

Native MS shows the highly cooperative nature of the two TTAI-binding events as the singly bound state remains a minor solution species as TTAI concentration increases. The IM-MS data at low collision energies indicate that binding of TTAI, especially the second copy, limits the conformational sampling of the protein as evidenced by the narrowing of the CCS distributions. The analysis of the CIU experiments further reveals the differences in the inherent potential for dynamic changes between apo-, single TTAI-bound, and double TTAI-bound α_1_-antitrypsin. The binding of two TTAI molecules stabilizes the protein in a ternary complex by favoring the population of compact conformers relative to more extended states such as those previously defined by IM-MS as polymerogenic intermediates.^44^,^45^

Integration of these findings with *B*-factor analysis of the high-resolution X-ray crystallographic structural data supports a model in which TTAI binds sequentially within s4A. In this model, the first TTAI peptide anneals to the more labile “breach” region at the base of the reactive loop between strands 3 and 5 of β-sheet A (s3A, s5A).^42^,^46^–^48^ In addition, the data show that IM-MS is a highly sensitive reporter of conformational changes in α_1_-antitrypsin even when these result in relatively small changes in overall CCS area of the molecule.

Combining the NMR and X-ray crystallographic data also dissects discrete structural changes from dynamic effects caused by peptide binding to α_1_-antitrypsin at the residue-specific level. Comparison of the similarly high-resolution crystal structures of native (1.8 Å; PDB ID: 3NE4^47^) and TTAI-saturated α_1_-antitrypsin shows that only β-sheet A undergoes substantial structural change. However, in solution, TTAI binding significantly perturbs the backbone chemical shifts for the majority of residues in the protein, with the exception of those within β-sheet C, which are relatively unaffected. This indicates that, in addition to the clear structural changes in β-sheet A seen in the crystal structure, TTAI binding in solution induces subtle but detectable changes throughout the rest of the protein. The reduction in native-state crosspeak intensities together with the concomitant emergence of discrete new peaks upon titration of TTAI indicates that the rate of exchange between the free and bound forms is slow relative to the associated chemical shift changes (≥ms timescale). The near-saturation of the protein at an equimolar ratio of TTAI shows the strength of the a1-antitrypsin–TTAI interaction, and is in agreement with the slow off-rate expected from the slow exchange rate that was observed in the NMR titration spectra. Interestingly, the well-dispersed crosspeaks arising from the TTAI-bound state appear to have narrower linewidths than those from the apo-state, suggesting that the binding of TTAI reduces the overall conformational lability of the protein, which is consistent with the increased kinetic stability revealed by the IM-MS data.

Taken together, the powerful combination of techniques used in these studies provides a wealth of useful information for developing TTAI-mimetic compounds that may be better suited to the development as marketable drugs. The multiplatform approach will similarly benefit the optimization of other lead compounds. The combination of NMR spectroscopy and X-ray crystallography provides unparalleled structural and dynamic information, but in a relatively labor-intensive process. IM-MS is a scalable, rapid method with low sample requirements that is suitable for automation. It provides direct, quantitative information upon conformational sampling of different species populated in solution. This is key to understanding a system characterized by dynamic conformational equilibria within- and between-distinct solution ensembles. It can report on the effects of peptide binding upon unfolding kinetics and probes how individual conformers are affected by peptide binding. Our data therefore support IM-MS as a primary screening tool in high-throughput drug development targeting disease-relevant protein behavior in α_1_-antitrypsin deficiency, and other disease mechanisms mediated by protein kinetic instability. Complementary methods focusing on IM-MS screen “hits” can then validate and improve understanding of the interactions in terms of conformational effects characterized at high resolution and their potential biological and therapeutic significance.

## Materials and Methods

### Protein biochemistry

Recombinant (including isotopically ^15^N-labeled material for NMR spectroscopy studies) and plasma-derived *M* and *Z* α_1_-antitrypsin were purified as described previously.^37^,^44^,^49^ Conformational homogeneity of the samples was confirmed by SDS-, nondenaturing, and TUG-PAGE and activity assay.^50^ TTAI tetrapeptide, modified by an acetyl group at the N-terminus and an amide group at the C-terminus, was purchased from Pepceuticals, United Kingdom. *T*_m_ values (mean from *n* = 3 experiments) were obtained by incubating 0.1 mg/mL of α_1_-antitrypsin in a thermal ramp as described previously.^51^

### X-ray crystallography

The α_1_-antitrypsin:TTAI ternary complex was formed by incubating purified recombinant α_1_-antitrypsin with TTAI at high concentration in a 2:1 molar ratio of 225 and 450 μ*M*, respectively, for 30 min at 25°C. Saturation of α_1_-antitrypsin with two copies of bound TTAI was confirmed by native MS studies. Crystals were grown by hanging drop vapor diffusion in a buffer of 26% w/v PEG3350 and 10 m*M* of Bis-Tris (pH 6.5). The resulting rod-like crystals were mounted in loops and cryocooled in cryoprotectant (26% w/v PEG 3350; 10 m*M* Bis-Tris [pH 6.5], 20% v/v glycerol). Synchrotron diffraction data were collected on beamline 23.2 at the ESRF, Grenoble, France (Table I). Data were processed using iMOSFLM^52^ and SCALA^53^ and solved by molecular replacement using PHASER.^54^ The coordinates of the native α_1_-antitrypsin crystal structure (PDB ID: 3NE4^47^) were used as the search model to solve the structure to a resolution of 1.92 Å. The structure of the ternary complex was deposited with the Research Collaboratory for Structural Bioinformatics (RCSB) PDB.

### NMR spectroscopy

Purified ^15^N-labeled α_1_-antitrypsin was prepared in 25 m*M* of Na_2_HPO_4_, 50 m*M* of NaCl, and 1 m*M* of EDTA, pH 8.0 buffer containing 10% v/v D_2_O, and 1% v/v DSS. Alpha_1_-antitrypsin was incubated at 37°C (urea, 0*M*) in the absence and presence of 10:1 molar equivalent of the tetrapeptide TTAI and NMR spectra recorded on the equilibrium states. All NMR data were recorded on a Bruker 700 Avance III spectrometer equipped with an HCN cryoprobe. 2D TROSY ^1^H-^15^N HSQC spectra were processed and analyzed using nmrPipe^55^ and CCPN^56^ software packages. All spectra were referenced to DSS at 0.0 ppm, manually phased, and baseline corrected. Spectra were analyzed using our previous assignment of the protein backbone (Biological Magnetic Resonance Data Bank accession number: 17804)^30^ by changes in native crosspeak intensity relative to the data on α_1_-antitrypsin in the absence of TTAI.

### Native and IM-MS

In brief, 15 μ*M* of α_1_-antitrypsin was incubated in protein purification buffer (37 m*M* NaCl, 2.7 m*M* KCl, 10 m*M* Na_2_HPO_4_, and 2 m*M* KH_2_PO_4_ [pH 7.4]) alone and in the presence of the TTAI tetrapeptide. Peptide:protein molar ratios of 0.1:1, 0.2:1, 0.3:1, 0.4:1, 0.5:1, 1:1, 3:1, and 5:1 were used and incubation was carried out for 3 days at 37.5°C.

Prior to MS experiments, samples were buffer exchanged using Micro Bio-spin P-6 Gel Columns (Biorad, United Kingdom) into 200 m*M* of ammonium acetate (pH 8). Protein was further concentrated and buffer exchanged using 10-kDa Amicon Ultra-0.5 Centrifugal Filters (Millipore, United Kingdom).

A first-generation Synapt traveling wave (T-Wave) IM mass spectrometer (Waters, Manchester, United Kingdom)^57^ was used for all native and IM measurements. Proteins were introduced into the mass spectrometer by means of nanoelectrospray ionization using borosilicate glass capillaries prepared *in-house*. Typical instrument parameters used were as follows: capillary voltage, 1.1 kV; cone voltage, 55 V; extraction cone voltage, 4.0 V; temperature, 40°C, trap CE, 6.0 V; transfer CE, 4.0 V; trap pressure, 1.5 mbar; bias, 4.0 V; backing pressure, 4 mbar. Calibration was performed using 33 μM of caesium iodide (Sigma-Aldrich), dissolved in high-performance liquid chromatography-grade water and acetonitrile, with formic acid (49:50:1, respectively).

For collision-induced dissociation experiments, the quadrupole was used to isolate the charge state of interest and fragmentation was performed in the trap TWave of the Synapt by increasing the trap collision voltage. For CIU, the same procedure was followed but this time IM separation was performed as well. For IM separations, the bias was increased to 19 V and the IM TWave velocity and wave height were set to 300 m/s and 7 V, respectively.

Data were acquired using MassLynx v4.1 (Waters). Arrival time distributions were converted to CCS by fitting to a power equation^58^ using the software Amphitrite.^59^ A set of standards with known CCS values^60^ were used and these were Bovine Serum Albumin, β-lactoglobulin A, and concanavalin A, all purchased from Sigma. Amphitrite was also used to plot CIU curves. ATD peak fitting was performed using the program Fityk 1.2.9.^61^ Peaks were modeled as Gaussian and fitted to the ATDs using the Levenberg–Marquardt algorithm.

**Table 1:** Crystallographic Data Parameters

*Data collection*	
Space group	C2
Unit-cell parameters	
*a,b,c* (Å)	113.71, 38.96, 93.13
*α*,*β*,*γ* (°)	90, 100.13, 90
Resolution limits (Å)	55.87–1.92 (2.02–1.92)
*No. of reflections*	
Total	118,452 (17,774)
Unique	30,573 (4467)
Completeness (%)	98.8 (99.2)
Multiplicity	3.9 (4.0)
Mean *I*/δ*I*	9.5 (2.5)
*R*_merge_	0.062 (0.408)
*Refinement*	
*R*_factor_	0.2184
*R*_free_	0.2400
No. of protein atoms	2896
*rmsd*	
Bonds (Å)	0.0127
Angles (°)	1.587
*Average B-factors (Å^2^)*	
Main chain	54.2
Side chain	56.7
TTAI 1st copy	37.1
TTAI 2nd copy	50.9
*C. Ramachandran criteria (%, assessed by MolProbity*‡*)*	
Ramachandran favored	93.68
Ramachandran outliers	1.37

Davis IW, Leaver-Fay A, Chen VB, Block JN, Kapral GJ, Wang X, et al (2007) MolProbity: all-atom contacts and structure validation for proteins and nucleic acids. Nucleic Acids Res35:W375–W383.
