# Supplementary figures and images for: An integrative approach combining ion mobility mass spectrometry, X-ray crystallography, and nuclear magnetic resonance spectroscopy to study the conformational dynamics of α1-antitrypsin upon ligand binding
Source: Protein Sci. 2015 Jul 14;24(8):1301–12. doi: 10.1002/pro.2706 (PMC4534181; doi:10.1002/pro.2706)

Supplementary figure 1

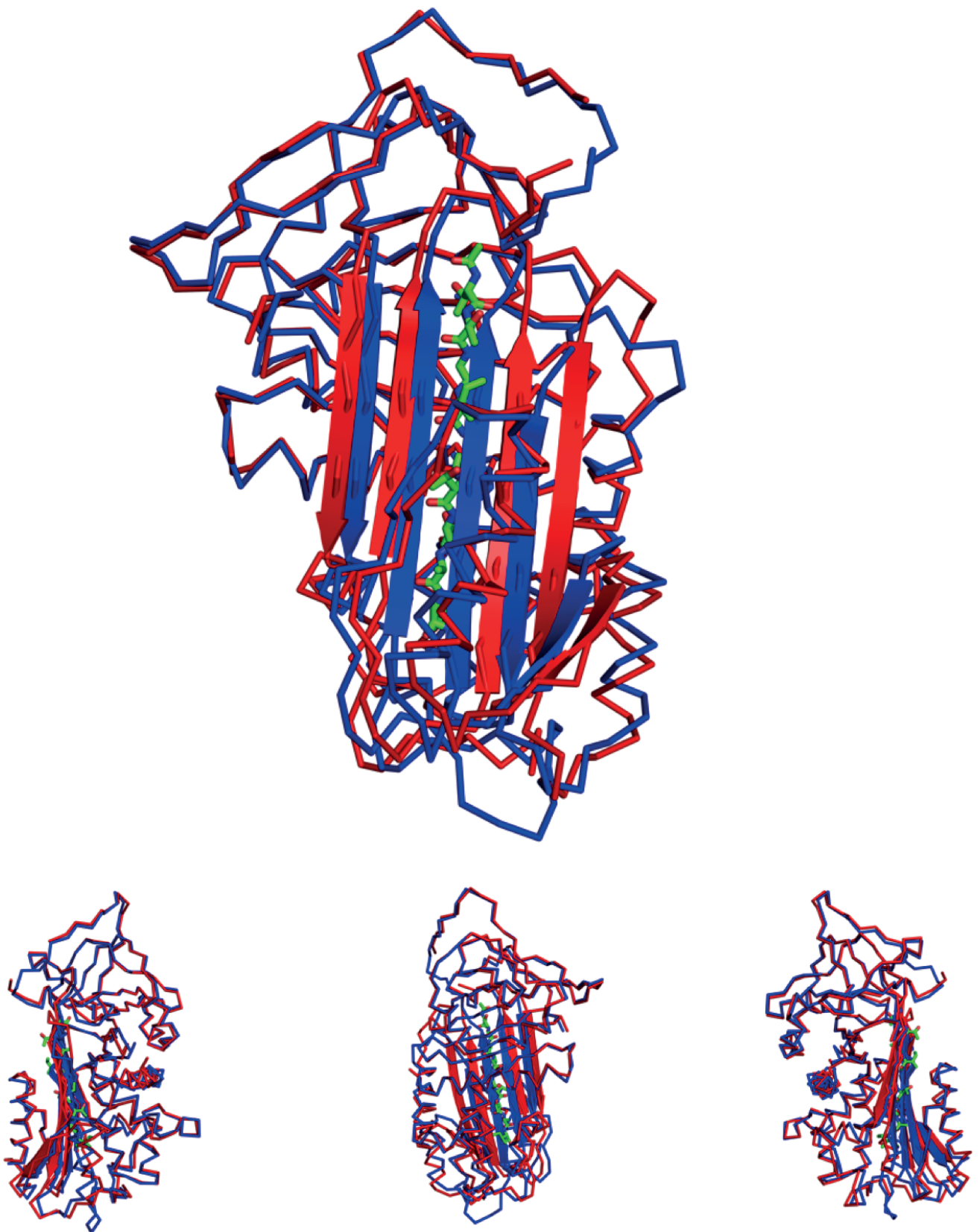

Supplementary figure 2

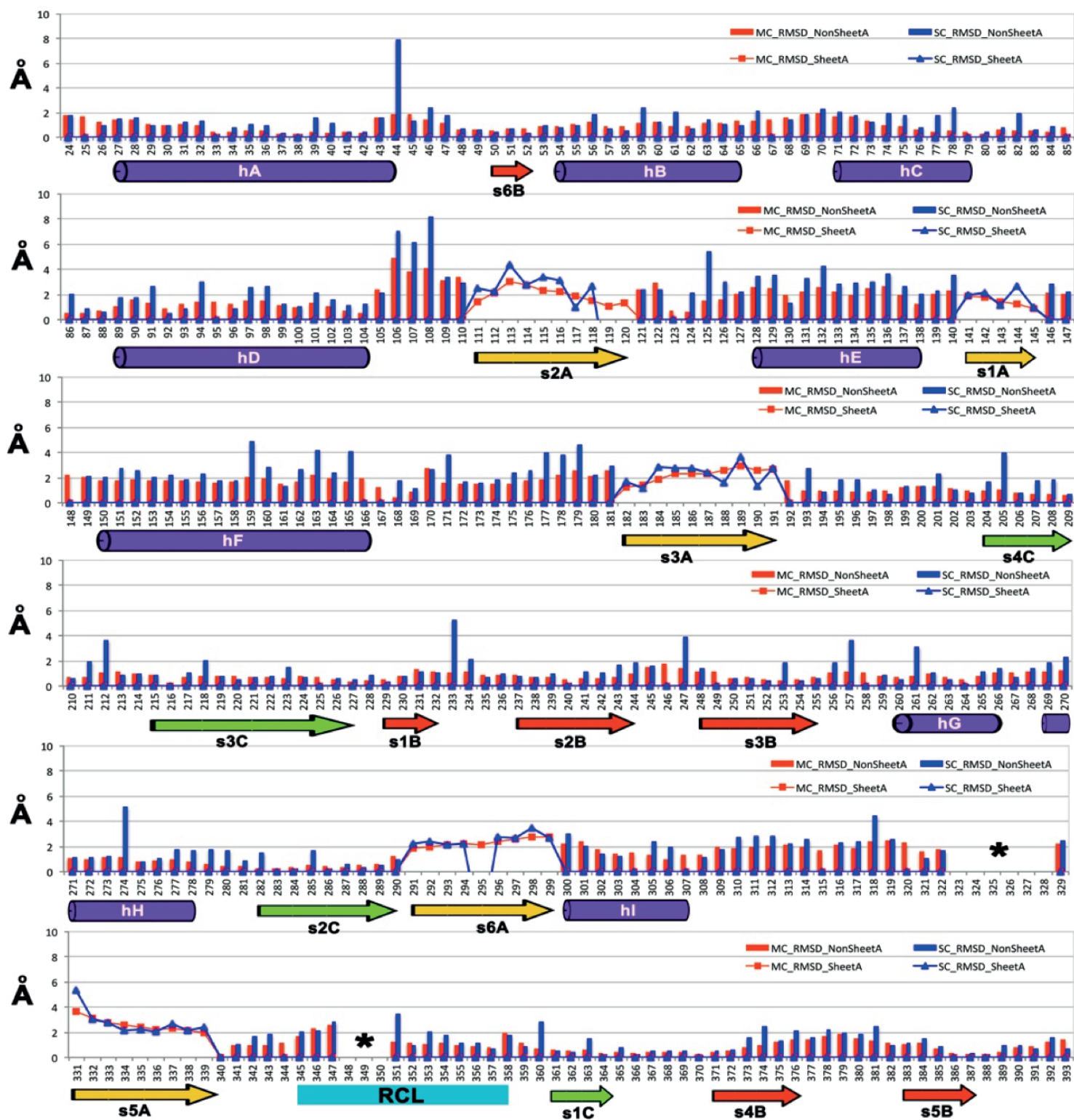

Supplementary figure 3

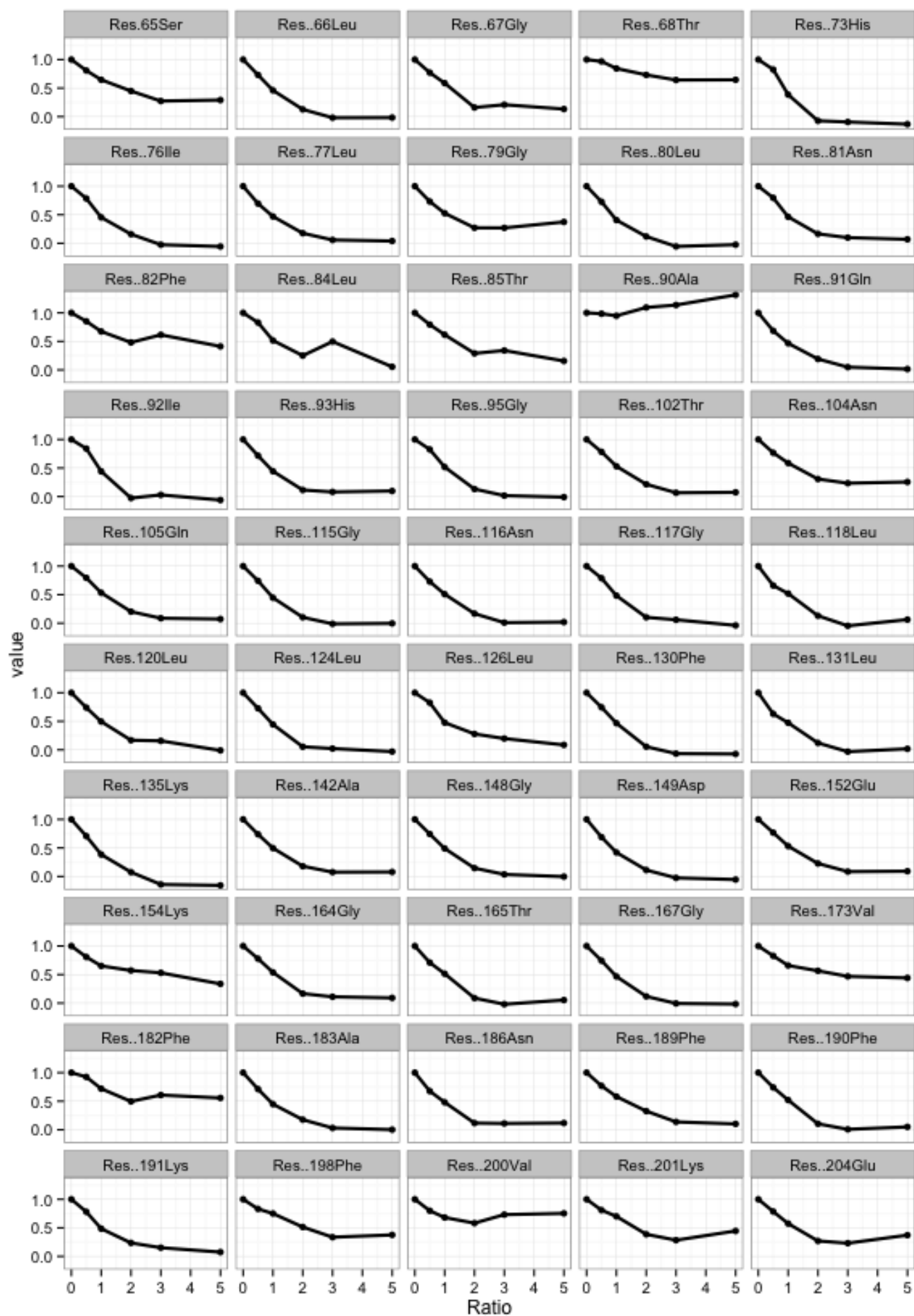

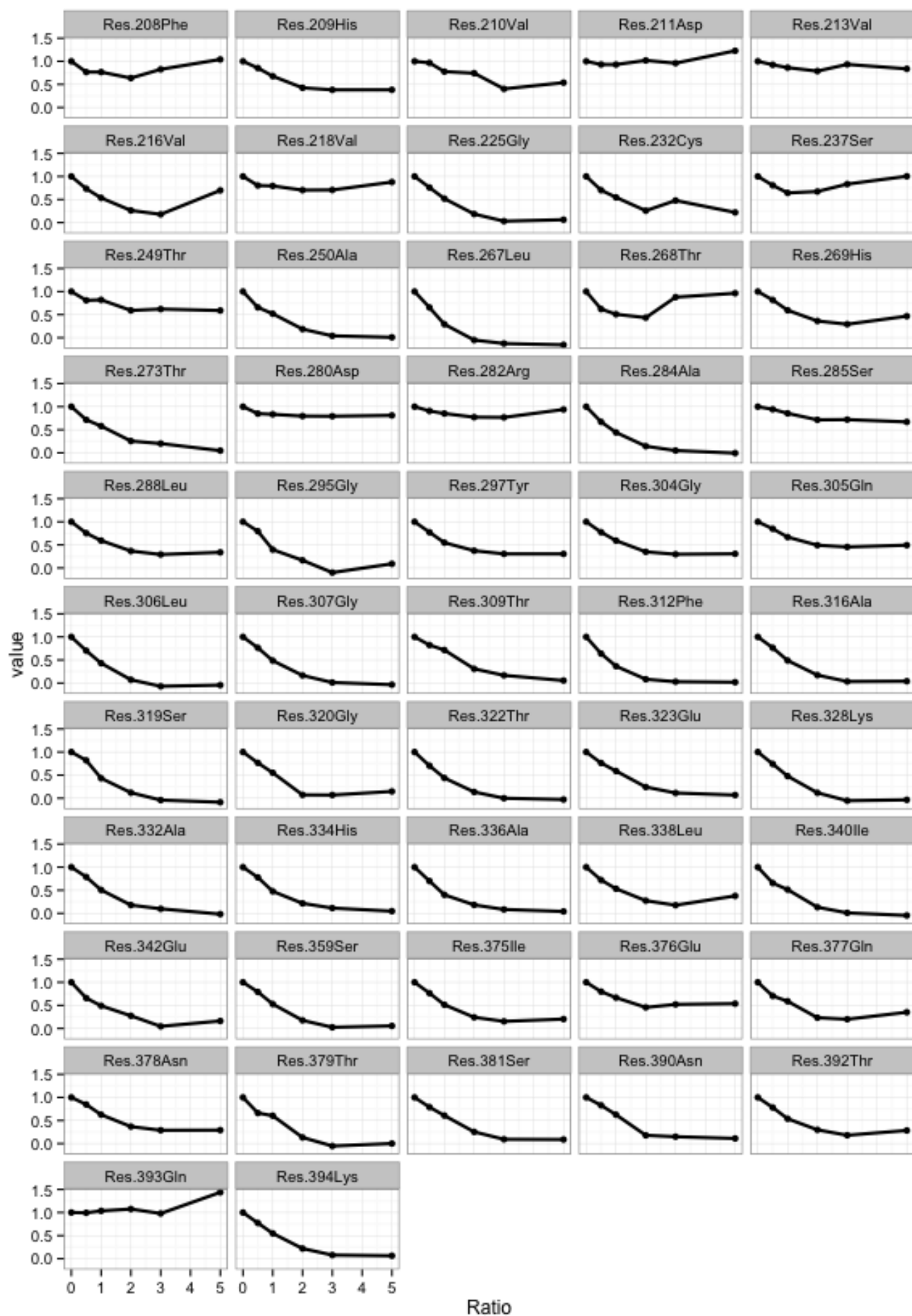

0.5:1

Supplementary figure 4

3:1

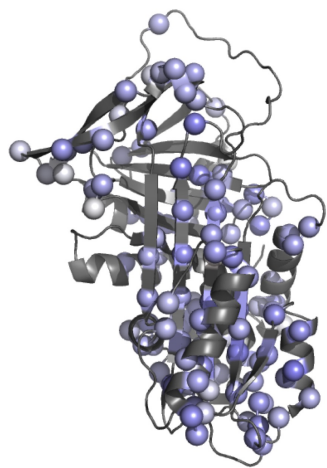180°  
↺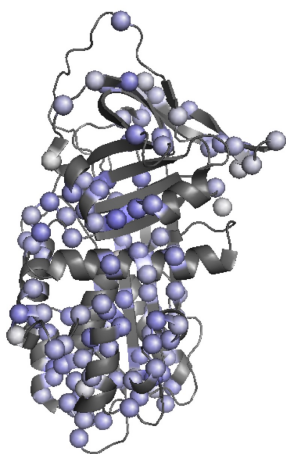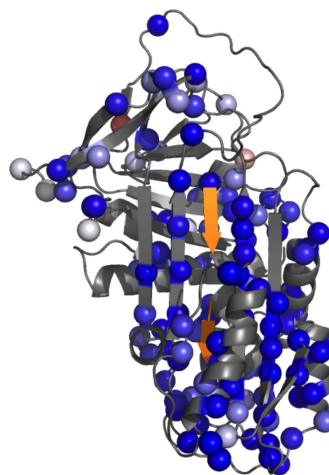180°  
↺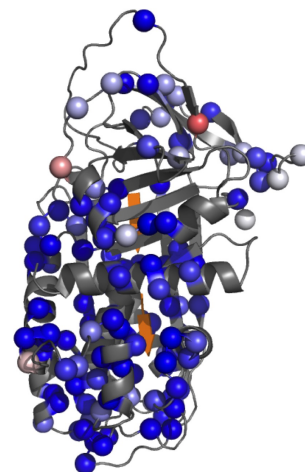

1:1

5:1

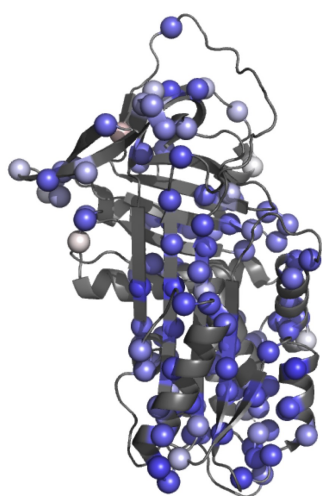180°  
↺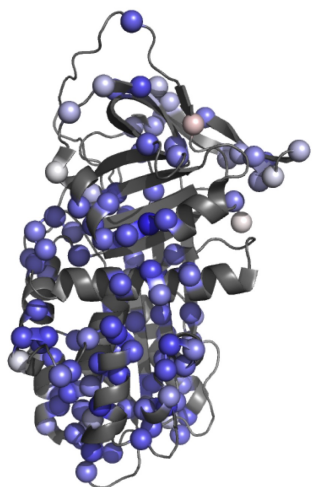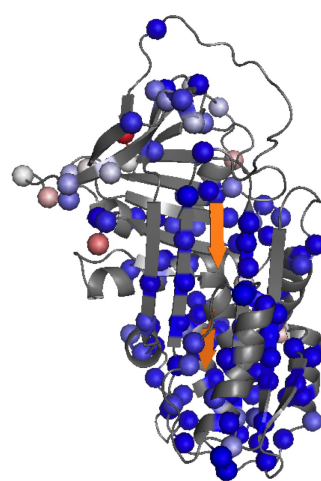180°  
↺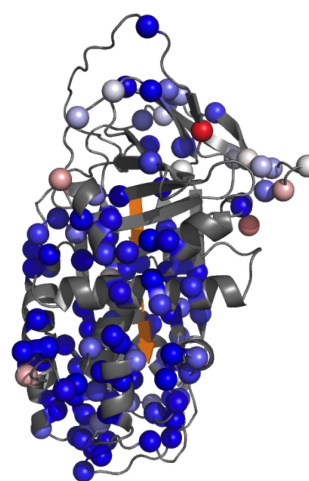

2:1

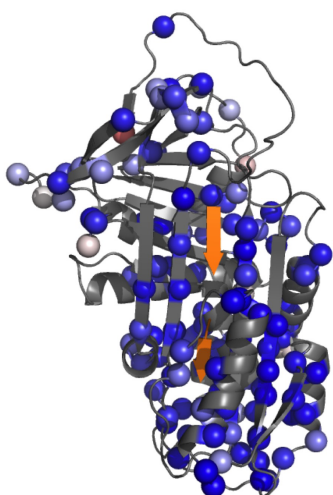180°  
↺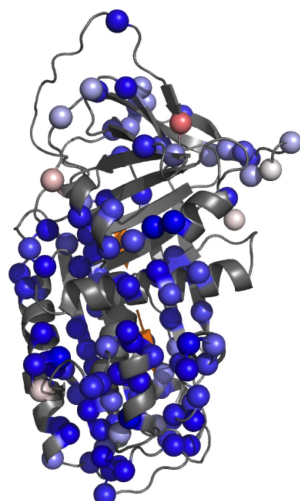

Supplement: Supplementary file 1 [file pro0024-1301-sd1.pdf]
